# Treatment of Croup

**Published:** 1856-06

**Authors:** 


### CROUP AND ITS METHOD OF TREATMENT.

Dr. Honerkopff has recently published a paper, in which he extols the administration of the sulphate of copper in this disease. He has used this substance in ninety-nine cases of croup, seventy-seven of which recovered; and the total quantity administered by him to these patients was 2846 grains, or, on an average, 31½ grains each. He has never seen any poisonous effects result from its use, although one child got twenty-seven grains daily for a week, or, in all, 216 grains; and another, 4½ years old, took 150 grains in seven days, and a third, aged 2½, 120 grains in three days. No inflammation, gangrene, or other bad symptoms took place. In eight out of the thirteen cases which proved fatal, there were either other diseases coexisting, or the author was called to see them too late, so that he considered that his remedy failed in only five out of the remaining eighty-two cases. The mode in which he administered the salt was to dissolve from six to eight grains in ℥j of water; and, according to the age of the patient, and the severity of the vomiting induced, to administer, more or less frequently, from a teaspoonful to a tablespoonful of this solution, until vomiting occurred. The nature of the vomiting should always regulate its administration; for the induction of vomiting is absolutely essential for the therapeutic action of the remedy, as it has a kind of specific influence on the disease. The more severe the case, the more frequently does the author administer the doses of the solution—giving them first at intervals of from ten to fifteen minutes. After from four to six doses have been given, sooner or later the urgent symptoms are found to abate. At first, indeed, after the first spoonful has been taken, the cough becomes less frequent and less troublesome, and the difficult respiration and general distress are also relieved. Thereafter, the croupy sound of the cough becomes changed, and crepitating mucous rales are audible. When this improvement has fairly commenced, the medicine may be administered less frequently, say every twenty or thirty minutes, and still more seldom as the malady recedes. In very severe cases the remedy should be continued every two hours, even after all traces of the cough have gone, because otherwise a relapse may occur in the evening, and necessitate a renewal of large doses of the salt. It is only in very bad cases, when, after a period of twelve or more hours, no change in the croup-sounds has taken place, that we need despair of success; and the author observes, that very often a change for the better takes place quite suddenly and unexpectedly. He warns us against remissness in our treatment after signs of improvement have commenced, as the disease, if unheeded, may break out afresh.

In cases where the copper fails to produce vomiting, we need expect no good from its administration, and we have then to choose between tracheotomy, cold effusion, and musk, and the two latter remedies, according to Honerkopff, may induce irritability of the stomach, and thus prepare the way for the renewed use of the sulphate..—*Journal fur Kinderkrankheiten*, March and April, 1855.

Dr. J. Samter, of Posen, has also written a paper in praise of the sulphate of copper in this disease. His remarks on its advantages and mode of administration so closely resemble those of Honerkopff, which we have given, that it is unnecessary to quote them here at length. He uses a solution of from four to eight grains, (and in severe cases, ten to twelve grains) in ℥ij of water; of this he gives ʒij repeatedly, till vomiting is induced, and thereafter ʒj every two hours.

In addition to the use of sulphate of copper, this author, at the beginning of the disease, applies four, eight, or twelve leeches to the larynx, and especially if there be any pain felt there, and he afterwards applies stimulating epithenes. He also uses the inhalation of powdered alum and sugar, employs warm baths, and envelops the feet in hot sand, etc.—*Gunsburg Zeitschrift*, vi. 2, 1855.

Dr. Luzsinsky considers that there are four therapeutical indications in the treatment of croup which must be attended to by the physician:

1st. To change the peculiar blood crasis which exists.

2d. To prevent the localization of the inflammatory process in the larynx.

3d. To treat the spasm of the larynx.

4th. To encounter and destroy the false membranes which have been formed.

For the fulfillment of the first indication, most men have recommended the use of mercurials; but Luzsinsky depends more upon alkalies, and especially on the carbonate of potass, which exercises a solvent action on all the albuminous products of the organism. Its use retards the development of the constituents of the blood, and greatly impairs the vital coagulability and inherent plasticity of that fluid. This is the theory of the action of this salt, which Eggert recommended as a specific in croup, after an experience of it in about 250 cases. It may be given advantageously in doses of from ℨss to ℨij daily. Carbonate of soda may do in mild cases, but the other alkali is alone to be relied on in more severe ones.

The second indication may be answered by the application of a blister, the size of a crown piece, to the upper part of the manobrium of the sternum.

Spasm of the larynx is most surely treated by opium, applied externally, in conjunction with vesicants (15 grains to ℨss of opium, to ℨss of lard) and also given in small doses internally.

To arrest the formation of the pseudo-membranes, nitrate of silver, in a concentrated solution, may be applied to the fauces and entrance of the larynx. Emetics may thereafter be given, and they are only necessary in the exudative stage. Luzsinsky gives decided preference to the use of sulphate of copper, which he administers by giving, every fifteen minutes, a teaspoonful of a solution of two or four grains (or even more) of the salt in ℨiiss of some fluid. He does not look upon tracheotomy, in croup in a favorable light.

